# Macroplastic and Microparticle Pollution in Beach Sediments from Urias Coastal Lagoon (Northwest Mexico)

**DOI:** 10.3390/toxics12060439

**Published:** 2024-06-18

**Authors:** Daniela Alvarado-Zambrano, José R. Rivera-Hernández, Carlos Green-Ruiz

**Affiliations:** 1 Engineering in Environmental Technology, Polytechnic University of Sinaloa, Km 3. Carretera Municipal Libre Mazatlan Higueras, Mazatlan 82199, Sinaloa, Mexico; dalvarado@upsin.edu.mx; 2 Mazatlan Academic Unit, Marine Sciences and Limnology Institute, Universidad Nacional Autónoma de México, Cap. Joel Montes Camarena, Mazatlan 82047, Sinaloa, Mexico; cgreen@ola.icmyl.unam.mx

**Keywords:** microplastic pollution, plastic debris, beach sediments, marine litter, microfiber

## Abstract

This study investigates the occurrence and characteristics of macroplastic and polymer microparticles in the Urias coastal lagoon’s beach sediments, in northwest Mexico. Coastal lagoons, productive and vulnerable ecosystems, are impacted significantly by anthropogenic activities, leadings to their pollution by various contaminants, including plastics. Our research involved sampling sediments from four sites within the lagoon that were influenced by different human activities such as fishing, aquaculture, thermoelectric power plant operations, industrial operations, and domestic wastewater discharge. Our methodology included collecting macroplastics and beach sediment samples, followed by laboratory analyses to identify the plastic debris’ size, shape, color, and chemical composition. The results indicated a notable presence of macroplastic items (144), predominantly bags, styrofoam, and caps made of polyethylene (PE), polypropylene (PP), and polyethylene terephthalate (PET). The polymer microparticles were mainly fibers, with cotton and polyester as the most common polymers, suggesting a significant contribution from clothing-related waste. The dominant colors of the microparticles were blue and transparent. High densities were observed in areas with slower water exchange. Our findings highlight the urgent need for better waste management practices to mitigate plastic pollution in coastal lagoons, preserving their ecological and economic functions.

## 1. Introduction

Coastal lagoons are considered one of the most productive ecosystems. They are shallow, semi-enclosed water bodies separated from the sea by barriers which are mainly sandy, but connected to it, permanently or temporarily, through one or more mouths [1]. These transitional ecosystems receive energy, sediments, solutes (e.g., nutrients and pollutants), and seawater/freshwater from oceans and continents. Depending on their geomorphology and communication with the sea (e.g., the number and length of their inlets), these water bodies protect the coasts against natural disasters and act as a nursery for larvae and juvenile organisms [2,3]. Because of the high residence time of particles and solutes, such as organic matter, nutrients, and pollutants, coastal lagoons are considered their temporal fate and act as a filter and a dispenser for them into adjacent open sea zones [4]. For these reasons, they are among the ecosystems most vulnerable to natural and anthropogenic pressure [5]. In addition to being thought of as supporting multiple ecosystem services [6], it is recognized that they have provided essential goods and services to humankind [2]. Several anthropogenic activities have developed around coastal lagoons, such as harbor and shipyard activities, seafood processing and oil storage, thermoelectric power plant activities, shrimp farming, etc. All these activities are potential sources of different contaminants (oil, potentially toxic elements, nutrients, organic matter, and plastic debris) [7,8].

Of these pollutants, plastics have become of interest. These materials are widely used, and their global production is forecasted to reach 1100 million tons by 2050 [9]. Waste mismanagement increases the plastic debris in aquatic environments, such as rivers, coastal lagoons, and the ocean [10,11,12]. 

Many of these plastics eventually end up in the ocean [10], where marine currents can transport them to areas where they were not directly generated [13].

These plastics degrade into smaller pieces through the physical stress provoked by abrasion, temperature, UV radiation, pH, and salt concentration [14]. Plastic pieces of less than 5 mm are known as microplastics, a term coined by Thompson et al. [15]. They are ubiquitous, since they have been found in marine water, sediments, organisms, air, and groundwater, among other locations [16,17,18,19].

The potential impacts of plastic pollution on marine organisms are a matter of great concern, even for the smallest organisms, such as marine microbes [20]. The sensitivity of some species over others to these pollutants depends on their characteristics, such as their feeding type, size, longevity, motility, habitat preferences, etc. [13], as well as the physical and chemical characteristics of the polymers [21].

Several authors have focused on plastic pollution in coastal lagoons. For instance, Abidi et al. [22] studied the occurrence of microplastics in sediments from the complex lagoon channel of Bizerte, Tunisia. They found a concentration range from 3000 to 18,000 particles/kg, with fibers as the most abundant shape and sewage discharges, fishing, and industrial production as the primary sources. Bayo et al. [23] investigated the occurrence, morphology, and chemical composition of microplastics in sediments from Spain’s Mar Menor coastal lagoon. They registered an average of 53.1 particles/kg, with low-density polyethylene (LDPE) as the most abundant polymer and fragments as the most abundant shape. Besides the runoff from water courses, tourism and fishing activities are recognized as its potential sources. Ragoobur et al. [4] studied the distribution of microplastics in water and sediments from four estuaries in Mauritius. They found that the concentration of microplastics in the sediments varied from 74 to 235 particles/kg, that 81% of the sedimented particles were fibers, and that 44% were identified as cotton–polyamide. Jayapala et al. [24] quantified the abundance of microplastics in sediments from mangrove ecosystems in Negombo Lagoon, Sri Lanka. They found that almost 10% of the mangroves’ surface was covered by single-use plastic debris, acting as “litter catchers”. They also mentioned that the debris’ occurrence positively correlates with the observed damage to seedlings and branches. 

**Figure 1:**
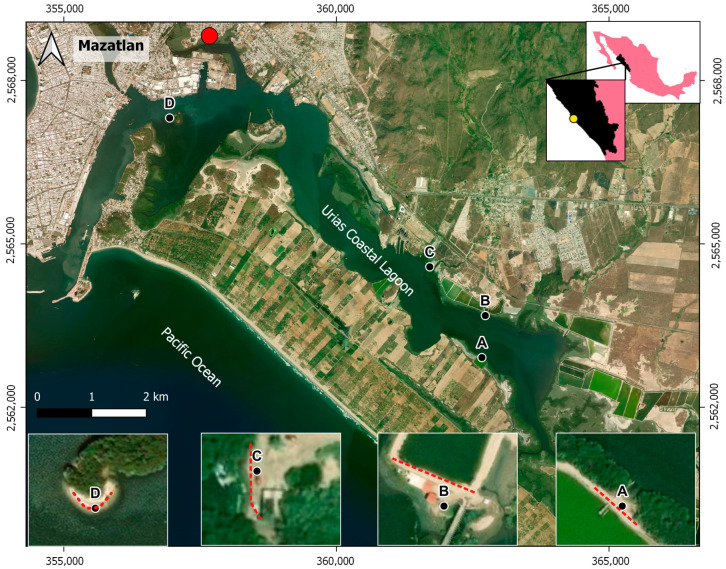
Urias coastal lagoon and sampling site (A, B, C, and D) locations. The yellow circle indicates the studied area location, the red circle indicates the “Urias II” wastewater treatment plant, and the red dotted lines are the sampling transects.

The Urias coastal lagoon, located at the margin of Mazatlán City (more than 500 000 inhabitants) in northwest Mexico (Figure 1), houses an industrial and fishing harbor as well as a thermoelectric power plant, shrimp farms, and food processing plants (tuna canning, shrimp packing, and a municipal slaughterhouse). In addition, this ecosystem receives water and litter discharges from the Jabalines stream through Estero del Infiernillo, which crosses a considerable portion of Mazatlán city, collecting rainwater runoff and irregular domestic sewage. In this respect, Rios-Mendoza et al. [16] evaluated the microplastic pollution in beach sediments and water from the tourist region of the Urias coastal lagoon (Mazatlan Bay). They included microplastic particles collected with a sediment trap from one site in this coastal lagoon. Despite the enormous amount of litter routinely observed in this water body, at present, this is the only study on microplastic carried out in the Urias coastal lagoon. The authors registered a mean flux of 515.4 particles/m^2^ each day and a concentration of 1088.8 particles/kg.

In this context, this study aims to determine the occurrence, physical characteristics (size, shape, and color), and chemical composition of the macroplastics and polymer microparticles (polymer microfibers or microfragments) in beach sediments from four internal sites of the Urias coastal lagoon. This research is crucial to understanding the extent and nature of the plastic pollution in this unique ecosystem.

## 2. Materials and Methods 

### 2.1. Sample Collection

In June 2023, four sampling sites on the margins of the Urias coastal lagoon were chosen based on their geology and accessibility (mangrove-free plain), and the influence of anthropogenic activities, for the collection of macroplastic and beach sediment samples (for microplastic analysis). Transects (35 to 70 m long; Table 1) along the high tide line were defined for each sampling site. All visible macroplastics were collected using a metallic tweezer and preserved in ziplock bags, and a triplicate of surficial beach sediment samples (depth: 1 cm; area: 25 cm × 25 cm) along each transect were collected using a stainless steel spoon and preserved in glass bottles for microfiber analysis. All samples were transported to the laboratory and stored at 4 °C until their respective analysis.

### 2.2. Sample Treatment and Analysis

#### 2.2.1. Macroplastic

Once in the laboratory, all the macroplastic samples were separated, cleaned, and rinsed using a brush and filtered purified water (MilliQ™, Sigma–Aldrich, Darmstadt, Germany), to remove the sand particles and organic matter adhered to the plastics’ surface. The color and shape of each item were recorded, and their chemical composition was determined with a Fourier Transformation Infrared spectroscope (Thermofisher Scientific™, Nicolet™ Summit™ (Waltham, MA, USA)).

#### 2.2.2. Microparticles

All beach sediment samples were dried in a laboratory oven (Blue M Electronic Company™, New Columbia, PA, USA) for 24–48 h, depending on the humidity, at 50 °C. According to Gimiliani et al. [25], 20 g of dried beach sediments were sieved using a series of four different-sized meshes (63, 250, 500, and 1410 μm) and filtered purified water (MilliQ^®^). For each fraction, sediments were recovered into porcelain capsules, dried at 50 °C for 24 h, and transferred to glass Petri dishes. Then, microparticles were isolated and characterized (by color and shape) using a stereomicroscope (ZEISS Stemi 508 stereo microscope with 5:1 zoom, Carl Zeiss AG, Oberkochen, Germany) and placed on glass fiber filters (0.7 μm Whatman 45 mm) for their subsequent chemical composition analysis using a micro–Fourier Transformation Infrared spectroscope (Thermofisher Scientific™, Nicolet™ iN™ 10 MX). 

### 2.3. Quality Control

To avoid plastic contamination, all the materials used during the sampling, treatment, and characterization were made of metal or glass. They were previously washed with filtered, purified water and oven-dried at 70 °C. In addition, the laboratory staff wore cotton clothes.

The entire sample processing was carried out in a laminar flow hood, and blanks were placed in glass Petri dishes and exposed to laboratory conditions to register any possible sample contamination with fibers present in the laboratory [25]. Three filters were exposed during digestion, isolation, and microscope visualization. No microplastics were detected. 

### 2.4. Statistics

For the microparticle data, descriptive statistics (average and standard deviation) of the triplicates at each sampling site were calculated using Microsoft Office Excel version 2405. A normality test was performed, and, since a non-normal distribution was obtained, a Generalized Linear Mixed Model analysis was performed to determine if there were differences among the mean microparticle concentrations of the sampling sites, using IBM SPSS (Statistic Package for Social Science) 25.0 statistic software.

### 2.5. Impact Assessment

The Anthropogenic Microparticles Pollution Index (AMPI) and the Coefficient of Anthropogenic Microparticles Impact (CAMI), suggested by Bouzekry et al. [26] (who modified them from Rangel-Buitrago et al. [27]), were employed in order to assess the impact of anthropogenic microparticles on each sampling site. For that, the following equations were used:

AMPI = ∑AMs/Surveyed area(1)

CAMI = Specific AMs shape/∑AMs (2)

where AMs refers to the sum of the amounts of microparticles in the three sampled quadrants for each sampling site; the Surveyed area represents the sum of the quadrant areas (3 transects × 0.25 m × 0.25 m = 0.1875 m^2^); and the Specific AMs shape is the amount of microparticles with a specific shape (such as fibers). Two sets of calculations were computed: one for all the anthropogenic microparticles, including anthropogenic cotton fibers (ACFs), and the other one excluding ACFs.

Once the calculations were performed, the sampling sites were categorized according to the nomenclature suggested by Rangel-Buitrago et al. [27].

## 3. Results and Discussion

### 3.1. Macroplastic

**Figure 2:**
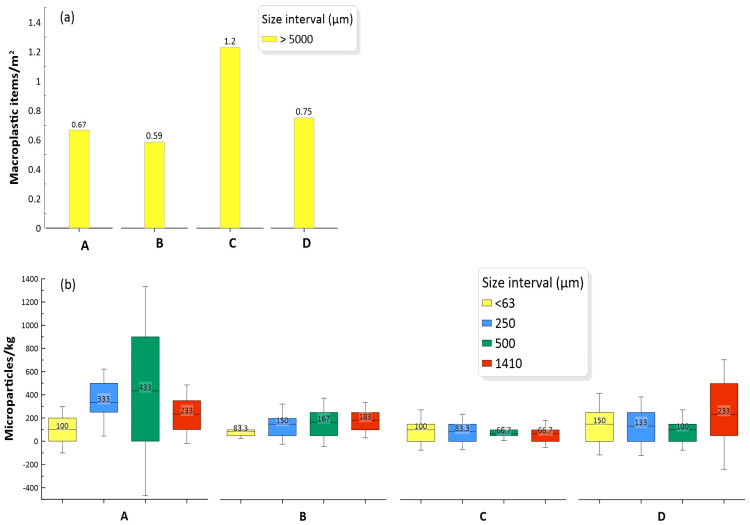
Macroplastic (**a**) and polymer microparticle (**b**) concentrations by size at sampling sites from Urias coastal lagoon. The letters A, B, C, and D indicate the sampling sites.

Overall, 144 macroplastic items were found across all four sampling sites (Table 2), with a dominance of bags (24.3%), styrofoam (18.1%), and caps (15.3%). The rest of the items had different uses (as bottles, foam, pipeline, sacks, nets, strips, wires, ropes, spoons, cups, clothespins, and other unidentified items). The highest concentration was found at site C (1.2 items/m^2^), which is influenced by fishing and aquaculture activities, as well as a thermoelectric power plant and the discharge of a little stream coming from a human settlement (Figure 2a; Table 1). This concentration and those of the other three sampling sites (0.59 to 0.75 items/m^2^) were higher than those found in sediments from the Ria Formosa lagoon, Portugal (0.01 to 0.17 items/m^2^) [28], and similar to those from the Qurum Natural Reserve lagoon (0.8 items/m^2^), a RAMSAR site on the Omani coast [29].

**Figure 3:**
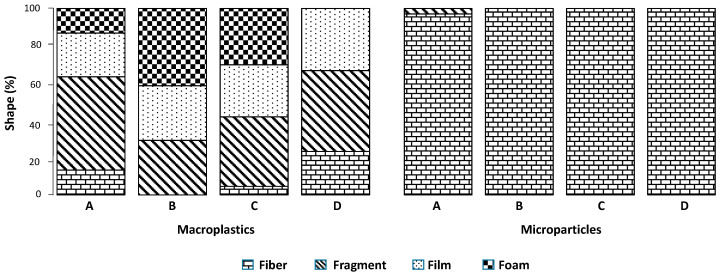
Macroplastic and microparticle shapes in sampling sites from Urias coastal lagoon. The letters A, B, C, and D indicate the sampling sites.

Fragments are the most representative shape of the macroplastic litter, with a significant occurrence of foam and film (Figure 3). There is no foam at the navigation channel (site D), where high hydrodynamic energy is present, but there is an increase in fiber concentration (>20%).

**Figure 4:**
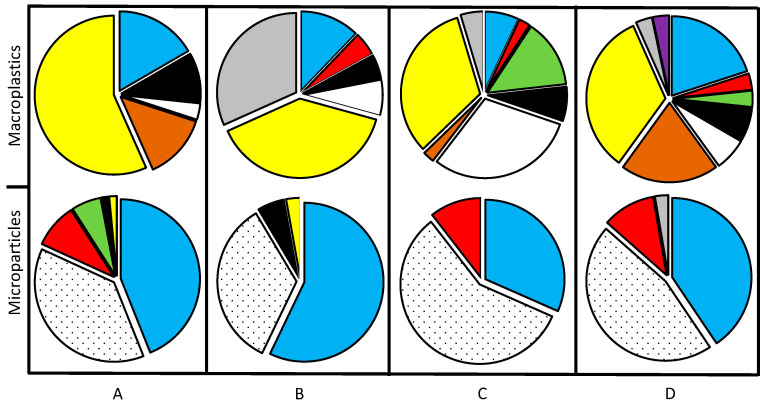
Macroplastic and microparticle colors in sampling sites from Urias coastal lagoon. The letters A, B, C, and D indicate the sampling sites. The colors in the graphics represent the particle colors, and the dotted area represents the transparent particles.

Yellow is the dominant color of the macroplastic items from all four studied sites (Figure 4), followed by gray, blue, and brown in the internal area of the coastal lagoon (sites A and B). An increase in color variability was observed at sites C and D, where a variety of potential anthropogenic plastic sources were observed, in addition to higher hydrodynamic energy.

**Figure 5:**
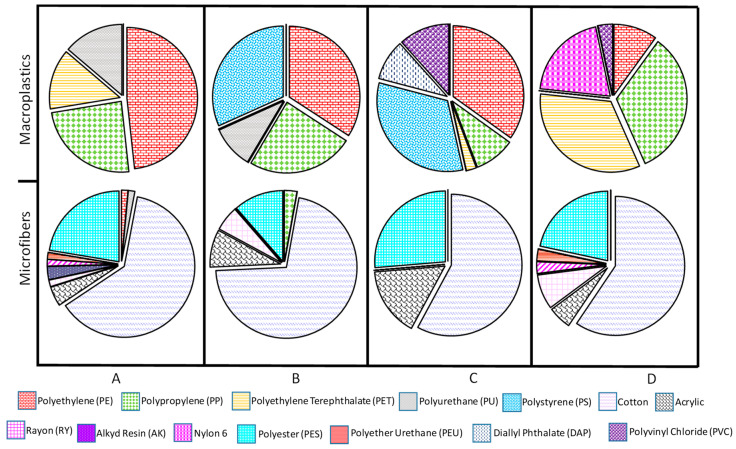
Chemical characterization of macroplastics and microparticles in sampling sites from Urias coastal lagoon. The letters A, B, C, and D indicate the sampling sites.

The dominant polymer was identified as polyethylene (PE), followed by polypropylene (PP) and polyethylene terephthalate (PET) (Figure 5). Site D, located at the navigation channel, is the only one showing the occurrence of nylon 6 as a result of the release of this polymer from fishing lines.

### 3.2. Microparticles 

Polymer microparticles were detected at all sites, with no significant differences among them (*p* > 0.05). Site A had an average of 1, 100 particles/kg (from 350 to 1850 particles/kg), while there were 583 particles/kg (from 450 to 700 particles/kg) in site B, 317 particles/kg (from 150 to 450 particles/kg) in site C, and 617 particles/kg (from 550 to 650 particles/kg) in site D. The high concentration of microparticles at site A (Figure 2) could be due to the influence of aquaculture activities and municipal wastewater discharges, as well as the slower water exchange in that sampling site area; according to Montaño-Ley et al. [30], there is a cyclonic eddy in this section of the lagoon that retards the flushing of pollutants, with an estimated residence time of 5–7 days [31].

The site with the second-highest microparticle concentration (site D) is within an area with fishing, port, and maritime transport activities. Another interesting characteristic of these two sites is the higher density of mangrove forests, which act like a marine litter tramp [32]. 

Other studies carried out on superficial sediments from coastal lagoons show a more significant amount of microparticles [22,33] (Table 3). Our results are similar to the ones presented by Ragoobur et al. [4], who analyzed the microplastics in four estuaries across Mauritius Island. Although it is difficult to make comparisons due to the lack of standardization of microplastic analysis techniques and concentration units, we decided not to remove natural polymer particles (cotton), which makes these differences more marked. The decision to include cotton particles is related to the idea that they also have an anthropogenic source; they are most likely derived from washing clothes.

In terms of shape, sites B, C, and D contained only fibers. In contrast, site A contained 97% fibers, and 3% of its microparticles were fragments (Figure 3). The average occurrence of fibers in the entire set of samples is 99% ± 1.52%, a high value similar to others that have been reported [4,34,35].

Regarding polymer colors, seven were found in the sediment samples (Figure 4); the most abundant color was blue, followed by transparent, red, green, black, yellow, and gray. These results agree with the review by [35] Garcés-Ordóñez et al. (2022) of 50 coastal lagoons worldwide. The sum of blue and transparent microparticles represents 82, 91, 89, and 86% of the microparticles at sites A, B, C, and D, respectively. 

Finally, with respect to the chemical composition of the polymers, the most abundant was cotton, followed by polyester (Figure 5). The combination of cotton and polyester makes up at least 81% of each sample’s net composition. Other polymers found in the samples were acrylic, rayon (RY), nylon 6, polyether urethane (PEU), polypropylene (PP), alkyd resin (AK), polyethylene (PE), and polyurethane (PU), in descending order.

Polyester is one of the most common polymers found in sediments, as was reported in a global review in 2015 by Van Cauwenberghe et al. [36] and, more recently, by Da-rabi et al. [37]. This leads to polyester being one of the polymers most consumed by fish [38,39].

The low variety of polymers and the high percentage of cotton and polyester seen could reflect a common source of these materials. In this case, these two main polymers are closely related to clothing; as Sillanpää and Sainio [40] and Statista [41] mentioned, textile fiber production worldwide is led by cotton and polyester, and these materials can reach aquatic environments through the machine-washing process [40,42]. There is a wastewater treatment plant (Urias II) discharging into the Urias coastal lagoon, which may be the primary source of these fibers.

Cotton fibers, as a textile, are commonly treated with dyes (which are used to color them and as additives) and flame retardants. These compounds are harmful to organisms, including humans, as they can bioaccumulate and biomagnify through the food chain. Their toxic effects include harm to the kidneys, lungs, skin, reproductive system, and other parts of the body [43,44,45].

As mentioned before, an impact assessment was carried out. In both cases, those including and not including the anthropogenic cotton fibers detected in beach sediments from the Urias coastal lagoon, there is a very high abundance of microparticles (>25 MPs/m^2^), with fibers having an extreme impact (>0.81) as a very dominant shape at site A and the only shape found at sites B, C, and D (Table 4). These data, in terms of microparticle classification, are similar to those registered by Bouzekry et al. [26], Abulouah et al. [46], and Ben-Haddad et al. [47]. They are different from those mentioned by Rangel-Buitrago et al. [27] and Arias et al. [48].

## 4. Conclusions

This study conducted in the Urias coastal lagoon, Mexico, reveals a significant presence of both macro- and microplastics in the sediments. The results indicate that the most common macroplastics are bags, styrofoam, and caps, while the most abundant polymer microparticles are cotton and polyester fibers. The high concentration of these fibers is mainly attributed to human activities such as the discharge of treated domestic wastewater.

This study compares the results from the Urias coastal lagoon with other global studies, noting that the abundance and types of plastics found are consistent with those reported in other coastal lagoons around the world, and it emphasizes the urgency of implementing effective strategies to control and mitigate plastic pollution in aquatic ecosystems, promoting the development of more advanced technologies for wastewater treatment.

## Figures and Tables

**Table 1:** Sampling site locations and their general characteristics.

	Sampling Sites			
	A	B	C	D
Geographical coordinates	23°10′10.1″ N	23°10′35.1″ N	23°11′3.9″ N	23°12′31.3″ N
	106°20′30″ W	106°20′28″ W	106°21′4.2″ W	106°23′52.9″ W
Sampling transects	45 m	70 m	35 m	40 m
Replicate	Each 15 m	Each 10 m	Each 10 m	Each 25 m
Surrounding activities	- Fishing - Aquaculture	- Fishing - Aquaculture	- Fishing - Aquaculture - Thermoelectric	- Dock - Industrial - Fishing - Human settlement - Recreation and tourist
General characteristics	- Low hydrodynamic energy - Low depth - Fine sand sediment - Mangrove area	- Low hydrodynamic energy - Low depth - Fine sand sediment - Mangrove area	- Low hydrodynamic energy - Low depth - Black fine sand sediment - Stream discharge - Mangrove area	- High hydrodynamic energy - Navigation channel for large vessels - Fine sand sediment

**Table 2:** Total (items) and relative (%) abundance of macroplastic litter by category at sampling sites from Urias coastal lagoon.

Category	Use	Sampling Sites				Relative Abundance	Polymer *
		A	B	C	D		
Bags	Chips, candies, rice, garbage, cookies, and groceries	5	11	12	7	24.3	PET, PP, and PE
Styrofoam		0	13	13	0	18.1	PS
Caps	Water, soda, and yogurt	6	7	3	6	15.3	PET, PP, and PE
Bottles	Water and soda	4	3	1	4	8.3	PET, PP, and PE
Foam		4	4	0	0	5.6	PU
Pipelines		0	0	6	1	4.9	PVC
Sacks		2	1	0	3	4.2	PP
Nets		0	0	0	6	4.2	Nylon 6
Strips		3	0	1	1	3.5	PP and PE
Wires		0	0	4	0	2.8	DAP
Ropes		1	0	1	0	1.4	PP and PE
Spoons		1	1	0	0	1.4	PE
Cups		1	0	1	0	1.4	PP and PE
Clothespins		0	0	0	1	0.7	PP
Other		3	1	1	1	4.2	PP and PE

* PET = polyethylene terephthalate; PP = polypropylene; PE = polyethylene; PS = Polystyrene; PU = polyurethane; PVC = Polyvinyl chloride; DAP = Diallyl phthalate.

**Table 3:** Occurrence of polymer microparticles in superficial sediments from coastal lagoons in different parts of the world.

Study Area	N	Abundance	Polymers *	Reference
Lagoon of Bizerte, Northern Tunisia	12	Min–max: 3.4 ± 0.2–18 ± 2.0 particles/g DW Average: 7.96 ± 6.84 particles/g DW	Not available	[22]
Mar Menor, Spain	17	Min–max: 8.2 ± 0.6–166.6 ± 1.7 particles/kg DW Average: 53.1 ± 7.6 particles/kg DW	LDPE, HDPE, PVE, PP, PS, Nylon, and PES	[23]
Lagos Lagoon, Southwest Nigeria	80	Min–max: not available Average: 43 ± 6.6 MPs/m^2^	PE and PP	[33]
Laguna de Términos, Southern Gulf of Mexico, Mexico	9	Min–max: 22.7–513.9 particles/g DW Average: not available	PE, POD, PET, SBR, PEO, and PVC	[34]
Four estuaries across Mauritius Island	36	Min–max: 0.0–734 particles/kg DW Average: 74–235 particles/kg DW	Cotton-PA, PE, PVA, PP, EVA, PET, and Nylon	[4]
Urias coastal lagoon, Mexico	12	Min–max: 317 ± 153–1100 ± 750 particles/kg DW Average: 654 ± 326 particles/kg DW	Cotton and PES	This study

N = Number of samples; * Polymers: PE = polyethylene; POD = polyoxadiazole; PET = polyethylene terephthalate; SBR = Styrene butadiene rubber; PEO = polyethylene oxide; PVC = Polyvinyl chloride; LDPE = low-density polyethylene; HDPE = High-density polyethylene; PVE =Polyvinyl ester; PP = polypropylene; PS = Polystyrene; PES = polyester; Cotton-PA = Cotton–polyamide; PVA = Polyvinyl alcohol; EVA = Ethylene-vinyl acetate; PUR = polyurethane.

**Table 4:** Anthropogenic Microparticles Pollution Index (AMPI) and Coefficient of Anthropogenic Microparticles Impact (CAMI) scores of beach sediments from Urias coastal lagoon.

Site	AMPI (MPs/m^2^)	Abundance	CAMI	Impact
A	352 *	Very high abundance	0.97 *	Extreme impact
	128	Very high abundance	0.92	Extreme impact
B	187 *	Very high abundance	1.00 *	Extreme impact
	53	Very high abundance	1.00	Extreme impact
C	101 *	Very high abundance	1.00 *	Extreme impact
	47	Very high abundance	1.00	Extreme impact
D	197 *	Very high abundance	1.00 *	Extreme impact
	80	Very high abundance	1.00	Extreme impact

* Including anthropogenic cotton fibers (ACFs).

## Data Availability

The data presented in this study are available on request from the corresponding author.
